# Impact of Thermocycling and Artificial Aging on the Flexural Strength and Fracture Resistance of CAD/CAM Dental Ceramics: A Systematic Review and Meta-Analysis

**DOI:** 10.3390/dj14080513

**Published:** 2026-08-12

**Authors:** Joyce Michel Mora Bayas, Luis Chauca Bajaña, Carol Ortiz Bustamante, Andrea Ordoñez Balladares, Laly Viviana Cedeño Sánchez, Ricardo Saltos Noboa, Diana Carolina Sandoval Benalcázar, Thalia Gabriela Alvarez Centeno, Ivonne Carrion Bustamante, Carlos Salazar Minda, David Alfredo Flor Ronquillo, Byron Velasquez Ron

**Affiliations:** 1 School of Dentistry, University of Guayaquil, Guayaquil 090101, Ecuador; bayasmichel045@gmail.com (J.M.M.B.);; 2 School of Dentistry, Universidad Católica de Santiago de Guayaquil (UCSG), Guayaquil 090101, Ecuador; 3 Universidad Politécnica Salesiana (UPS), Sede Guayaquil 090105, Ecuador; 4 School of Dentistry, Universidad Bolivariana del Ecuador, Durán 092406, Ecuador; 5 Carrera de Odontología, Universidad de Las Américas (UDLA) Ecuador, Quito 170102, Ecuador

**Keywords:** CAD/CAM ceramics, thermocycling, artificial aging, flexural strength, fracture resistance, zirconia, lithium disilicate, systematic review, meta-analysis

## Abstract

**Background:** CAD/CAM dental ceramics are widely used for indirect restorations because of their favorable esthetic properties, biocompatibility, and mechanical performance. However, long-term exposure to thermal fluctuations, moisture, and cyclic loading may compromise their structural integrity. This systematic review and meta-analysis evaluated the effect of artificial aging protocols on the mechanical properties of CAD/CAM dental ceramics. **Methods:** A systematic search was conducted in PubMed, Embase, Scopus, Web of Science, LILACS, Google Scholar, and ProQuest from inception to June 2026. In vitro studies evaluating CAD/CAM ceramics subjected to thermocycling and/or artificial aging protocols were included. The primary outcomes were flexural strength and fracture resistance. Random-effects meta-analyses were performed using standardized mean differences (SMDs) or pooled means with 95% confidence intervals (CIs). Subgroup analyses were conducted according to ceramic category, and sensitivity analyses were performed using a leave-one-out approach. **Results:** Five studies comprising 16 independent comparisons were included in the flexural strength meta-analysis, while five studies contributed 11 comparisons to the fracture resistance analysis. Artificial aging protocols significantly reduced the flexural strength of CAD/CAM ceramics (SMD = −1.68; 95% CI: −2.28 to −1.08; *p* < 0.00001). The greatest reduction was observed in zirconia-based ceramics (SMD = −3.31), followed by hybrid/PICN ceramics (SMD = −1.37) and lithium disilicate/lithium silicate ceramics (SMD = −1.25). Feldspathic and glass-based ceramics showed no significant reduction. The pooled fracture resistance after aging was 1312.85 N (95% CI: 1131.59–1494.10 N), indicating that most CAD/CAM ceramics maintained clinically relevant fracture resistance despite aging. **Conclusions:** Artificial aging protocols adversely affect the flexural strength of CAD/CAM dental ceramics, particularly zirconia-based materials. Nevertheless, fracture resistance generally remains within clinically acceptable ranges. Material composition plays a critical role in determining susceptibility to aging-related degradation, highlighting the importance of ceramic selection for long-term restorative performance.

## 1. Introduction

The widespread adoption of computer-aided design and computer-aided manufacturing (CAD/CAM) technologies has significantly transformed restorative dentistry by enabling the fabrication of highly accurate, esthetic, and durable ceramic restorations. CAD/CAM ceramics, including lithium disilicate, zirconia-reinforced lithium silicate, feldspathic ceramics, and yttria-stabilized zirconia polycrystals (Y-TZP), are currently among the most frequently used materials for indirect restorations because of their favorable mechanical properties, biocompatibility, and esthetic performance [1,2,3,4,5].

Among these materials, lithium disilicate and zirconia have gained particular attention due to their excellent clinical outcomes and long-term survival rates. Lithium disilicate ceramics exhibit high translucency and satisfactory flexural strength, whereas zirconia-based ceramics demonstrate superior fracture resistance and toughness resulting from their unique transformation-toughening mechanism [5,6,7,8]. Advances in material science have also led to the development of highly translucent and multilayer zirconia systems designed to improve esthetics while maintaining adequate mechanical performance [9,10].

Despite these advantages, ceramic restorations are continuously exposed to a challenging oral environment characterized by thermal fluctuations, moisture, chemical degradation, and cyclic masticatory forces. These conditions may induce microstructural alterations and progressive degradation that compromise the long-term mechanical stability of restorative materials [11,12,13]. Since ceramics are inherently brittle materials, the presence and propagation of microscopic defects can significantly influence their resistance to fracture and clinical longevity.

Artificial aging protocols have been widely used in laboratory studies to simulate the effects of long-term clinical service. Among these methods, thermocycling is one of the most commonly employed protocols because it reproduces temperature variations associated with the intake of hot and cold foods and beverages. Repeated thermal stresses generated during thermocycling may promote crack propagation, hydrolytic degradation, and changes in the microstructure of ceramic materials [14,15,16]. In zirconia-based ceramics, exposure to moisture and temperature may additionally induce low-temperature degradation, a phenomenon associated with phase transformation and potential deterioration of mechanical properties over time [17].

Several investigations have evaluated the influence of artificial aging protocols on the mechanical performance of CAD/CAM ceramics, reporting outcomes such as flexural strength, fracture resistance, hardness, and fracture toughness. However, the available evidence remains inconsistent, with some studies reporting significant reductions in mechanical properties after artificial aging protocols, while others have observed limited or negligible effects depending on the ceramic composition and aging protocol employed [18,19,20]. Furthermore, the increasing diversity of CAD/CAM ceramic materials has complicated direct comparisons among studies.

Given the growing clinical use of CAD/CAM ceramics and the lack of consensus regarding the impact of artificial aging protocols on their mechanical behavior, a comprehensive synthesis of the available evidence is warranted. Therefore, the aim of this systematic review and meta-analysis was to evaluate the effect of artificial aging protocols on the flexural strength and fracture resistance of CAD/CAM dental ceramics. Secondary mechanical outcomes were summarized qualitatively when sufficient comparable data for quantitative synthesis were unavailable.

## 2. Materials and Methods

### 2.1. Protocol and Registration

**Figure 1:**
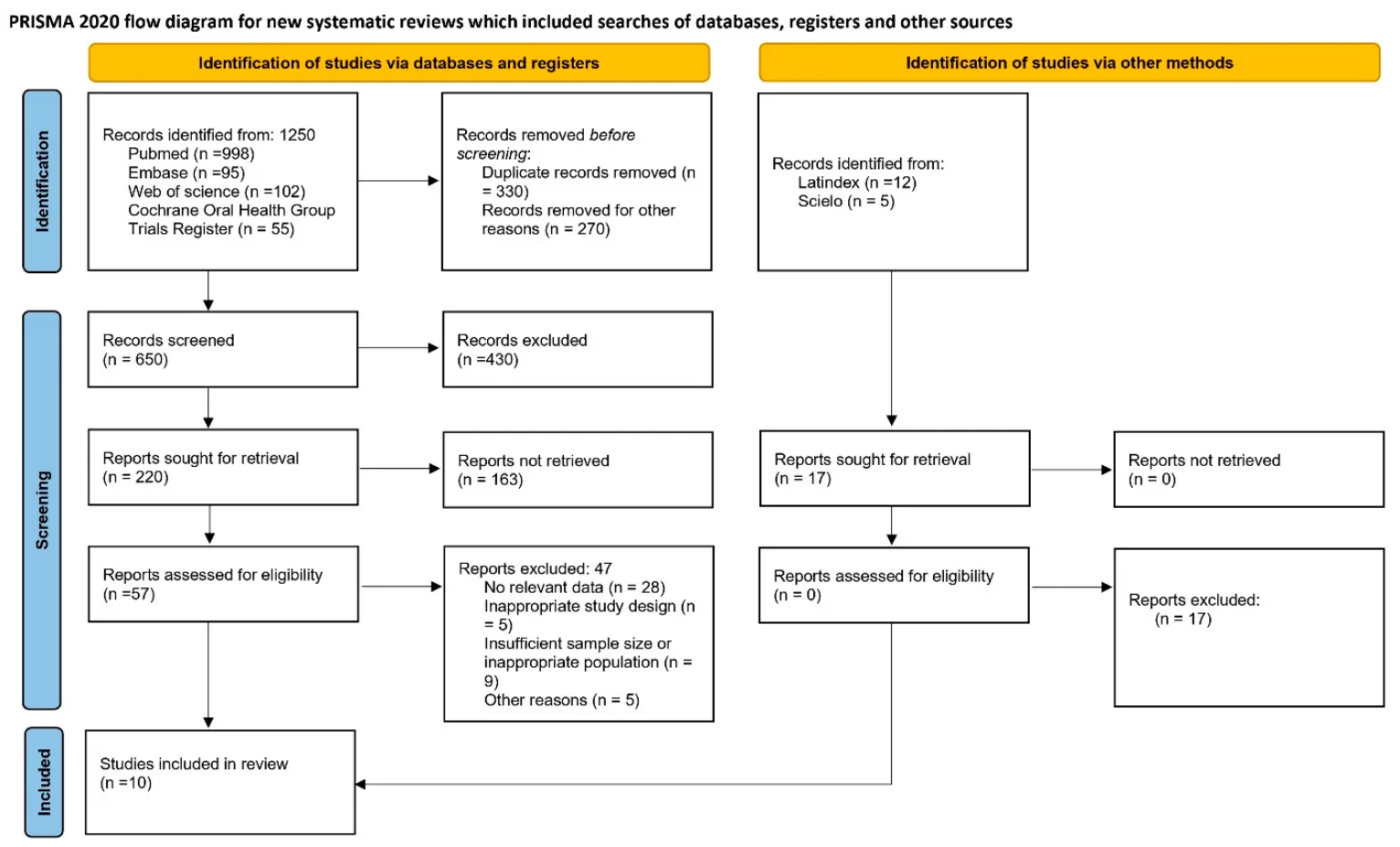
Flowchart of selected studies.

This systematic review and meta-analysis was conducted according to the Preferred Reporting Items for Systematic Reviews and Meta-Analyses (PRISMA 2020) guidelines (Figure 1). The review protocol was prospectively registered in the International Prospective Register of Systematic Reviews (PROSPERO) under registration number CRD420261430910 [21]. In addition, the completed PRISMA 2020 checklist is provided as Supplementary Material (File S1).

### 2.2. PICO Question

Do artificial aging protocols affect the mechanical performance of CAD/CAM dental ceramics?

P (Participants): In vitro studies evaluating CAD/CAM dental ceramics used for indirect dental restorations, including lithium disilicate ceramics, zirconia-based ceramics, zirconia-reinforced lithium silicate ceramics, feldspathic ceramics, glass ceramics, hybrid ceramics, and polymer-infiltrated ceramic network (PICN) materials.

I (Intervention): Thermocycling and/or artificial aging protocols, including hydrothermal aging, water storage, autoclave aging, mechanical fatigue, cyclic loading, chewing simulation, or other laboratory aging methods designed to simulate long-term clinical conditions.

C (Comparison): For flexural strength analyses, CAD/CAM ceramic specimens subjected to thermocycling and/or artificial aging were compared with non-aged control specimens or baseline measurements obtained before artificial aging protocols. For fracture resistance analyses, studies evaluating CAD/CAM ceramics after artificial aging protocols were considered eligible regardless of the presence of a non-aged control group.

O (Outcome): The primary outcomes were flexural strength (MPa), including conventional flexural strength and biaxial flexural strength measurements, and fracture resistance (N). Flexural strength outcomes were quantitatively synthesized using comparisons between aged and non-aged specimens whenever available. Fracture resistance outcomes were synthesized using data obtained after standardized artificial aging protocols, including thermocycling, hydrothermal aging, water storage, cyclic fatigue, and chewing simulation. Because different testing methods, restoration designs, ceramic compositions, and aging protocols were used across studies, pooled analyses were performed using standardized effect size measures when appropriate. Secondary outcomes included hardness (Vickers or Knoop hardness), fracture toughness (MPa·m½), elastic modulus (GPa), Weibull modulus, fatigue strength, survival rate, and other relevant mechanical properties, which were summarized qualitatively when quantitative synthesis was not feasible.

### 2.3. Information Sources and Search Strategy

A comprehensive electronic search strategy was developed and conducted on all available records from database inception to June 2026. The search was performed in five major electronic databases: PubMed (MEDLINE), Embase, Scopus, Web of Science, and LILACS (Latin American and Caribbean Health Sciences Literature). To minimize publication bias and identify potentially eligible unpublished studies, a complementary search of gray literature was conducted using Google Scholar and ProQuest Dissertations & Theses Global.

All retrieved references were imported into Zotero software (version 7.0; Corporation for Digital Scholarship, Fairfax, VA, USA) for bibliographic management and duplicate removal. Subsequently, article screening and selection were conducted using Rayyan QCRI (Qatar Computing Research Institute, Doha, Qatar), allowing independent assessment by reviewers during title/abstract and full-text screening phases.

The search strategy was developed according to the PICO framework and combined controlled vocabulary (MeSH and Emtree terms) with free-text keywords related to CAD/CAM dental ceramics, thermocycling, artificial aging, and mechanical properties. Boolean operators (AND, OR) were used to optimize sensitivity and specificity. Keywords included terms such as “CAD/CAM ceramics”, “dental ceramics”, “lithium disilicate”, “zirconia”, “zirconia-reinforced lithium silicate”, “glass ceramics”, “hybrid ceramics”, “polymer-infiltrated ceramic network”, “thermocycling”, “artificial aging”, “hydrothermal aging”, “autoclave aging”, “water storage”, “mechanical fatigue”, “cyclic loading”, “chewing simulation”, “flexural strength”, “fracture resistance”, “fracture toughness”, “hardness”, and “elastic modulus”.

In addition, a manual search was performed in relevant peer-reviewed journals specializing in prosthodontics, restorative dentistry, biomaterials, and dental materials science. The reference lists of all included studies and relevant reviews were also screened to identify additional eligible publications.

The complete PubMed (MEDLINE) search strategy was as follows:

((“Dental Ceramics”[Mesh] OR ceramic* OR “CAD/CAM ceramic*” OR zirconia OR “yttria-stabilized zirconia” OR Y-TZP OR “lithium disilicate” OR “zirconia-reinforced lithium silicate” OR ZLS OR “glass ceramic*” OR “hybrid ceramic*” OR PICN OR “polymer infiltrated ceramic network”)

AND

(“Thermocycling” OR thermocycl* OR “thermal cycling” OR “thermal aging” OR “artificial aging” OR aging OR ageing OR “hydrothermal aging” OR “autoclave aging” OR “water storage” OR “mechanical fatigue” OR fatigue OR “cyclic loading” OR “chewing simulation”)

AND

(“Mechanical Properties” OR “flexural strength” OR “fracture resistance” OR “fracture toughness” OR hardness OR “elastic modulus” OR “Weibull modulus”))

No restrictions regarding publication year were applied. Only studies published in English were considered eligible. Whenever available, filters for laboratory-based or in vitro studies were applied. The search syntax was adapted appropriately for each database according to its specific indexing system and controlled vocabulary.

### 2.4. Inclusion and Exclusion Criteria

Studies were considered eligible if they met the following inclusion criteria: (1) in vitro studies evaluating CAD/CAM dental ceramics used for indirect restorations; (2) studies investigating the effects of thermocycling and/or artificial aging protocols, including thermocycling, hydrothermal aging, water storage, autoclave aging, mechanical fatigue, cyclic loading, chewing simulation, or combinations thereof; (3) studies reporting quantitative mechanical outcomes such as flexural strength, fracture resistance, fracture toughness, hardness, elastic modulus, or Weibull modulus; (4) for flexural-strength analyses, studies including a non-aged control group or sufficient baseline data to permit quantitative comparison; for fracture-resistance analyses, studies reporting post-aging load-to-fracture data were eligible regardless of whether a non-aged control group was available; (5) studies reporting sample size and quantitative data allowing extraction or calculation of means and standard deviations for at least one outcome of interest; and (6) studies published in peer-reviewed journals in the English language.

The exclusion criteria were as follows: (1) studies that did not provide separate quantitative data for CAD/CAM ceramic materials; (2) studies that failed to specify the thermocycling or artificial aging protocol applied; (3) studies evaluating only surface treatments, glazing procedures, polishing techniques, staining methods, bonding protocols, or surface roughness without assessing the effects of aging on mechanical properties; (4) studies focused exclusively on marginal adaptation, color stability, translucency, optical properties, phase transformation analysis, microstructural characterization, or wear behavior without reporting mechanical outcomes; (5) studies involving materials other than CAD/CAM ceramics, such as resin composites, acrylic resins, metallic materials, or conventionally fabricated ceramics; (6) studies with duplicate datasets already reported in another included publication; (7) reviews, systematic reviews, meta-analyses, narrative reviews, letters to the editor, editorials, conference abstracts, conference proceedings, books, book chapters, technical reports, guidelines, case reports, case series, and expert opinions; and (8) studies for which the full text could not be obtained after contacting the corresponding authors on three separate occasions over a three-week period.

### 2.5. Study Selection Process and Data Extraction

Two investigators (CC and AO) independently performed the study selection and data extraction procedures using a standardized data collection form specifically developed for this systematic review. Any disagreements regarding study eligibility or extracted information were resolved through discussion and, when necessary, by consultation with a third investigator (BVR), who acted as an independent blinded reviewer.

The study selection process was conducted in two sequential phases. In the first phase, titles and abstracts retrieved from the electronic databases were screened to identify potentially relevant studies according to the predefined eligibility criteria. In the second phase, the full texts of all potentially eligible articles were independently assessed to determine final inclusion. Studies that did not meet the inclusion criteria or lacked sufficient methodological information were excluded. When essential data required for the quantitative synthesis were unavailable or unclear, attempts were made to contact the corresponding authors by email up to three times, with a one-week interval between requests, to obtain additional information.

For each included study, the following information was extracted: first author, year of publication, ceramic category, CAD/CAM ceramic material evaluated, sample size per group, mechanical outcome assessed, artificial aging protocol, testing methodology, and principal findings. Additional methodological variables included the type of aging procedure employed (thermocycling, water storage, mechanical fatigue, or thermomechanical aging), presence or absence of thermocycling and mechanical fatigue, number of aging cycles, loading conditions, flexural testing method (three-point bending or biaxial flexural strength test), fracture resistance testing protocol (load-to-fracture test), and testing standards or laboratory conditions used. Particular attention was given to extracting quantitative data related to flexural strength and fracture resistance, including mean values, standard deviations, and sample sizes for both aged and non-aged groups whenever available.

The characteristics of the studies included in the quantitative synthesis of flexural strength are presented in Table 1, whereas the detailed methodological and material-related variables for these studies are summarized in Table 2. Similarly, the characteristics of the studies included in the fracture resistance meta-analysis are presented in Table 3, and their detailed methodological information is reported in Table 4.

Although dual independent screening and data extraction were performed, inter-rater agreement statistics such as Cohen’s kappa could not be calculated retrospectively because individual reviewer screening records were not preserved. Nevertheless, all disagreements were resolved through consensus, ensuring the accuracy and consistency of the final dataset used for qualitative and quantitative analyses.

### 2.6. Risk of Bias Assessment

The methodological quality of the included in vitro studies was independently assessed by two reviewers (CC and AO) using the Risk of Bias tool for In Vitro Studies (RoB-Iv), which has been specifically developed to evaluate potential sources of bias in laboratory-based dental research. Any disagreements between reviewers were resolved through discussion and, when necessary, consultation with a third reviewer (BVR) until consensus was achieved.

The following domains were evaluated: (1) specimen preparation, including standardization of specimen fabrication and material processing; (2) randomization, referring to the allocation of specimens to experimental and control groups; (3) blinding of outcome assessment, when reported; (4) outcome measurement, including the adequacy and standardization of mechanical testing procedures; and (5) statistical analysis and reporting, considering the appropriateness of statistical methods and completeness of data presentation. Each domain was classified as “low risk of bias,” “some concerns,” or “high risk of bias,” according to the RoB-Iv criteria.

Particular attention was given to the standardization of artificial aging protocols, including thermocycling, water storage, mechanical fatigue, and thermomechanical artificial aging protocols, as well as to the consistency of mechanical testing methods used to evaluate flexural strength and fracture resistance. Studies were judged based on the transparency and reproducibility of specimen preparation, aging conditions, testing protocols, and statistical reporting.

The overall risk of bias for each study was determined based on the assessments across all domains. The results were graphically summarized using traffic light plots and weighted bar charts generated with the robvis package in R software, providing a comprehensive visualization of methodological quality across studies and risk-of-bias domains.

### 2.7. Statistical Analysis

#### 2.7.1. Qualitative Analysis

A qualitative synthesis was performed based on the extracted methodological and material-related variables summarized in Table 1, Table 2, Table 3 and Table 4. All included studies were in vitro investigations evaluating CAD/CAM dental ceramics subjected to thermocycling and/or artificial aging protocols. The materials analyzed included lithium disilicate, zirconia-based ceramics, zirconia-reinforced lithium silicate, feldspathic ceramics, glass ceramics, hybrid ceramics, resin nanoceramics, and polymer-infiltrated ceramic network (PICN) materials.

Flexural strength and fracture resistance were the primary outcomes evaluated. Flexural strength was assessed using three-point bending or biaxial flexural strength tests, whereas fracture resistance was mainly evaluated through load-to-fracture tests. Aging protocols varied across studies and included thermocycling, water storage, mechanical fatigue, cyclic loading, and thermomechanical aging. Due to methodological variability in ceramic composition, specimen geometry, number of aging cycles, loading conditions, and testing protocols, a qualitative synthesis was first conducted to identify patterns in material behavior before quantitative meta-analysis.

#### 2.7.2. Meta-Analysis

Quantitative synthesis was performed when at least three studies or independent comparisons reported comparable outcomes. Separate meta-analyses were conducted for flexural strength and fracture resistance. For flexural strength, aged CAD/CAM ceramic specimens were compared with non-aged control specimens or baseline values. For fracture resistance, data obtained after artificial aging protocols were synthesized when studies reported comparable load-to-fracture outcomes.

The two primary outcomes required different quantitative approaches because of differences in the available comparator data. For flexural strength, aged specimens were compared with corresponding non-aged controls, allowing estimation of the aging-related effect using standardized mean differences. For fracture resistance, comparable non-aged control groups were not consistently reported; therefore, post-aging load-to-fracture values were synthesized as single-arm pooled means expressed in Newtons. Accordingly, the fracture-resistance analysis was descriptive and represented the average post-aging performance under the experimental conditions evaluated. It should not be interpreted as a direct estimate of the effect of aging or of the change from an unaged condition.

Because the included studies evaluated different ceramic materials, aging protocols, testing methods, and measurement conditions, the standardized mean difference (SMD) with 95% confidence intervals (CI) was used as the summary effect measure. Negative SMD values indicated lower mechanical performance after thermocycling or artificial aging compared with non-aged controls. A random-effects model using the inverse-variance method was applied to account for expected methodological and material heterogeneity among studies.

Raw flexural-strength values obtained using three-point bending and biaxial tests were not directly combined as absolute MPa values. Within each material comparison, aged and non-aged specimens were evaluated using the same testing configuration. The standardized mean difference was calculated as the difference between the aged and non-aged means divided by the pooled within-comparison standard deviation, with a small-sample correction corresponding to Hedges’ g. Therefore, the resulting effect size represented the relative aging effect within each testing method rather than a direct conversion between three-point and biaxial flexural-strength values. Because standardization cannot completely remove differences in stress distribution and failure mechanics between testing configurations, a sensitivity analysis restricted to studies using three-point bending was also performed.

Artificial aging was considered an umbrella intervention encompassing different laboratory procedures intended to simulate one or more components of long-term intraoral exposure. For the flexural-strength analysis, aged specimens were compared with their corresponding non-aged controls, and the pooled standardized mean difference represented the average direction and magnitude of change across the aging protocols evaluated. The random-effects model allowed the true effect to vary across studies and did not assume equivalence among artificial aging protocols or exposure intensities. Protocol-specific subgroup analyses were not performed because too few independent studies were available within each aging category and because aging modality was closely associated with ceramic category and other study-level characteristics. Detailed information regarding the aging protocols is presented in Table 2 and Table 4.

Statistical heterogeneity was assessed using Cochran’s Q test, the I^2^ statistic, and Tau^2^ (τ^2^). I^2^ values were interpreted as low (<25%), moderate (25–50%), or high (>50%) heterogeneity. When data permitted, prediction intervals were calculated as exploratory measures to illustrate the potential dispersion of effects. However, because several analyses included only a small number of independent studies, these intervals were interpreted cautiously and were not considered precise estimates of the effects expected in future studies. Funnel plots were generated for exploratory visual assessment only. Given the limited number of studies and comparisons, they were not used to establish the presence or absence of publication bias or small-study effects, and formal tests of funnel-plot asymmetry were not interpreted.

Sensitivity analyses were performed using the leave-one-out method, in which each study or independent comparison was sequentially excluded to evaluate its influence on the pooled effect size and heterogeneity. In addition, when multiple material comparisons were extracted from the same study, multilevel random-effects meta-analysis was considered to account for the hierarchical structure of the data and avoid overestimating the precision of pooled estimates.

Subgroup analyses were conducted according to ceramic category when sufficient data were available. These included zirconia-based ceramics, lithium disilicate/lithium silicate ceramics, hybrid/PICN materials, and feldspathic or glass-based ceramics. Protocol-specific subgroup analyses were not conducted because the available number of independent studies within each aging category was insufficient to provide reliable estimates.

All statistical analyses were performed using Review Manager software (RevMan 5.4, The Cochrane Collaboration) and R software version 4.3.2 (R Foundation for Statistical Computing, Vienna, Austria), using the meta and metafor packages. A *p*-value < 0.05 was considered statistically significant.

## 3. Results

### 3.1. Risk of Bias Assessment

The methodological quality of the included studies was assessed using the Risk of Bias tool for In Vitro Studies (RoB-Iv). Overall, the included studies demonstrated satisfactory methodological quality, and no study was classified as having a high risk of bias.

Specimen preparation, outcome measurement, and statistical analysis were judged as low risk of bias in all studies (100%), reflecting adequate standardization of specimen fabrication, testing procedures, and statistical reporting. In contrast, some concerns were identified in the randomization domain in 60% of the studies because allocation procedures were insufficiently described. Blinding of outcome assessment represented the most frequently underreported methodological criterion, resulting in some concerns in all included studies.

Overall, four studies (40%) were classified as having a low risk of bias, whereas six studies (60%) were judged as presenting some concerns. Importantly, no study was considered to have a high risk of bias. Although methodological reporting deficiencies were identified, particularly regarding randomization and blinding procedures, the overall quality of the available evidence was considered acceptable for quantitative synthesis. A summary of the risk-of-bias judgments across all evaluated domains is presented in Table 5 and Table 6.

### 3.2. Overall Effect of Artificial Aging Protocols on Flexural Strength

A total of five studies comprising 16 independent comparisons were included in the quantitative synthesis. The random-effects meta-analysis demonstrated that artificial aging protocols significantly reduced the flexural strength of CAD/CAM dental ceramics compared with non-aged controls (SMD = −1.68; 95% CI: −2.28 to −1.08; *p* < 0.00001). The pooled effect size indicated a large detrimental effect of aging on the mechanical performance of ceramic materials.

**Figure 2:**
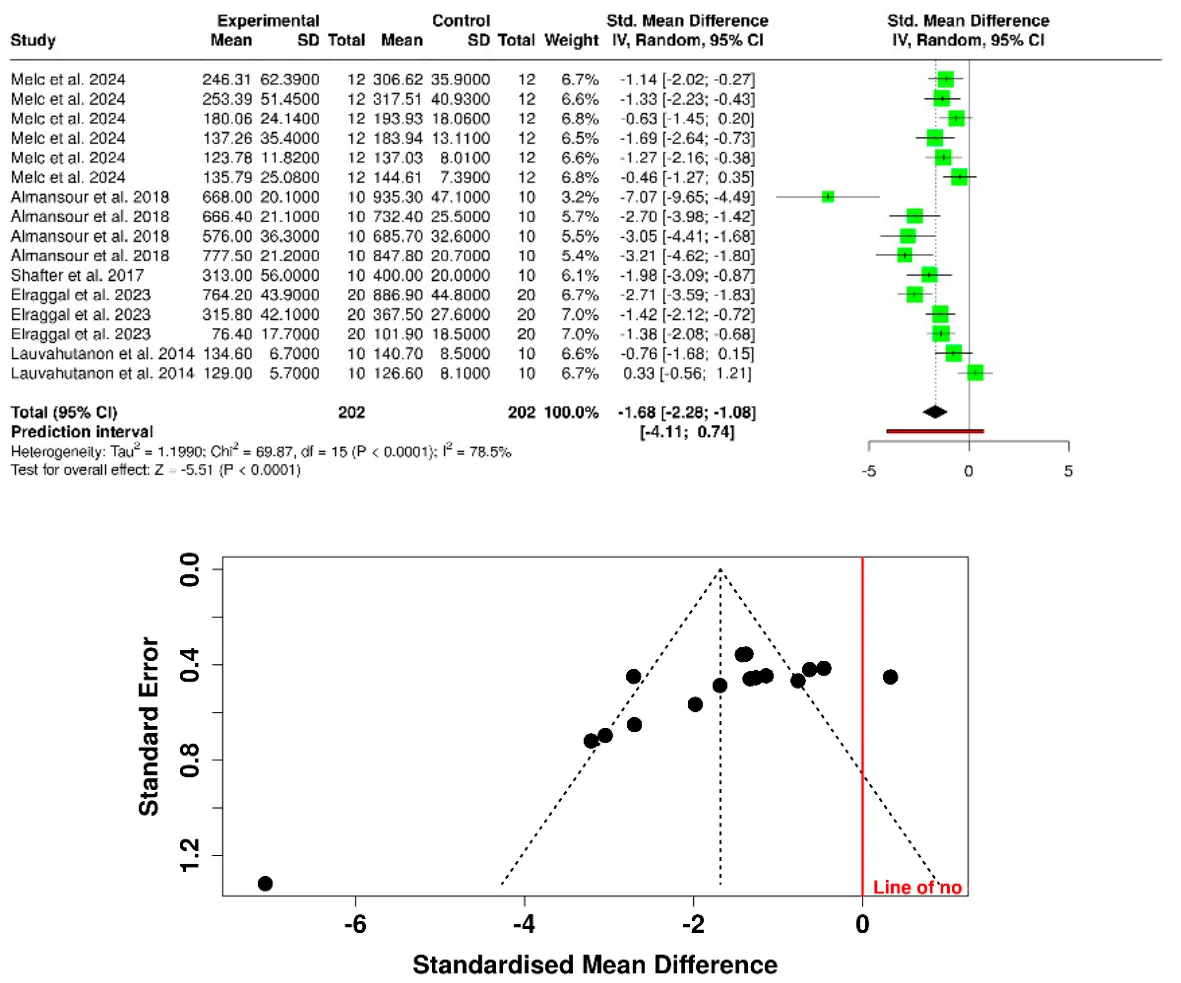
Overall effect of artificial aging protocols on the flexural strength of CAD/CAM dental ceramics. Forest plot showing the pooled standardized mean difference (SMD) in flexural strength between aged and non-aged specimens using a random-effects model. Negative values indicate lower flexural strength after aging. Funnel plot assessing potential publication bias and small-study effects among the included comparisons [22,23,24,25,26].

Substantial heterogeneity was observed among the included comparisons (I^2^ = 78.5%; Q = 69.87; *p* < 0.00001), suggesting variability related to differences in ceramic composition, aging protocols, and testing methodologies. Despite this heterogeneity, most studies consistently showed lower flexural strength values after artificial aging protocols, supporting the negative impact of artificial aging protocols on CAD/CAM ceramics (Figure 2).

### 3.3. Subgroup Analysis: Zirconia-Based CAD/CAM Ceramics

**Figure 3:**
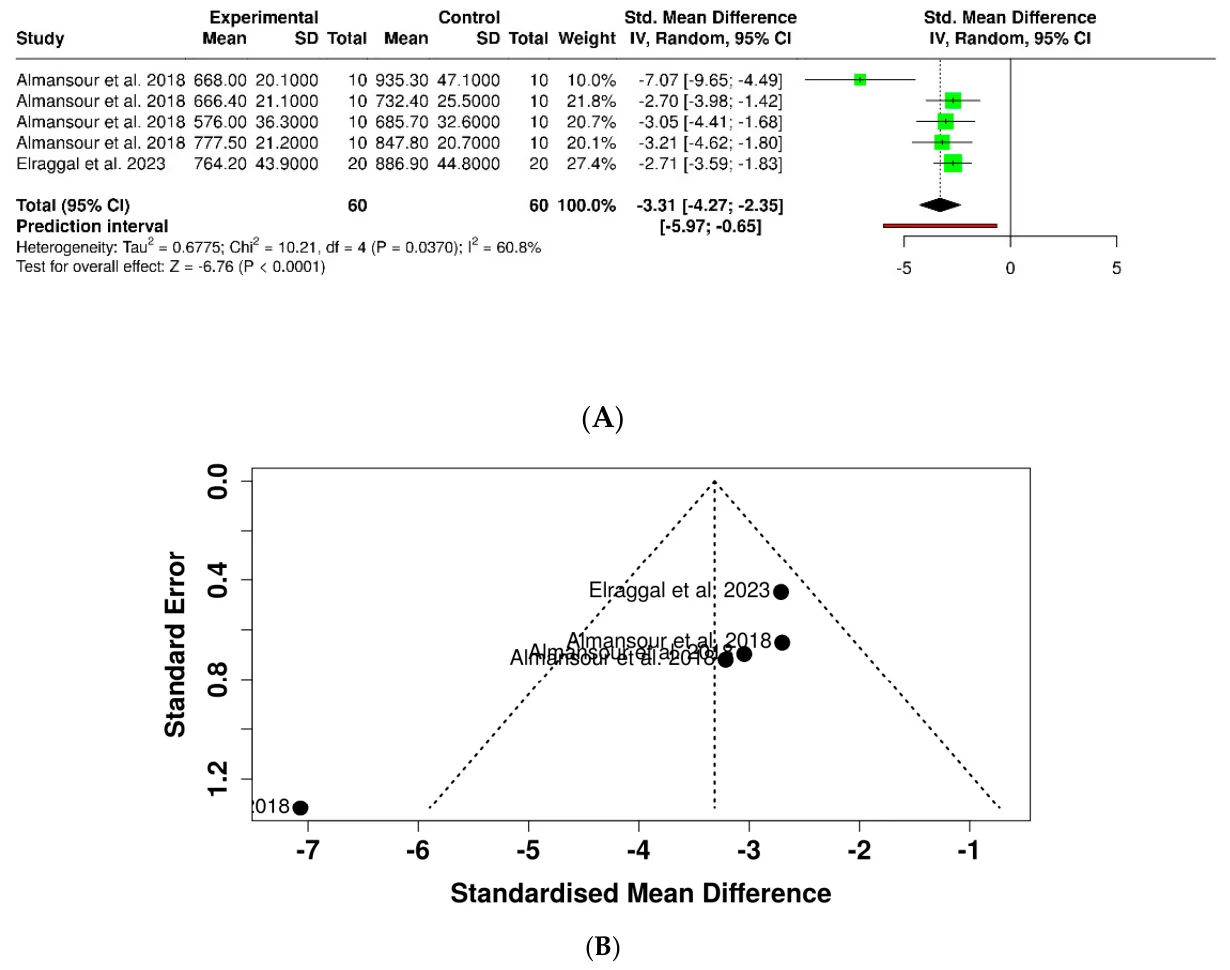
Subgroup meta-analysis of zirconia-based CAD/CAM ceramics. (**A**) Forest plot showing the pooled standardized mean difference (SMD) in flexural strength between aged and non-aged zirconia specimens using a random-effects model. Negative values indicate lower flexural strength after artificial aging protocols. (**B**) Funnel plot of the zirconia subgroup comparisons [24,25].

Five independent comparisons from two studies were included in the zirconia subgroup analysis. Artificial aging protocols significantly reduced the flexural strength of zirconia-based CAD/CAM ceramics compared with non-aged controls (SMD = −3.31; 95% CI: −4.27 to −2.35; *p* < 0.00001). Moderate heterogeneity was observed among studies (I^2^ = 60.8%; *p* = 0.037). The prediction interval remained below the null effect (95% PI: −5.97 to −0.65); however, because this estimate was based on only two independent studies, it should be regarded as exploratory and interpreted with caution (Figure 3).

### 3.4. Subgroup Analysis: Lithium Disilicate and Lithium Silicate Ceramics

**Figure 4:**
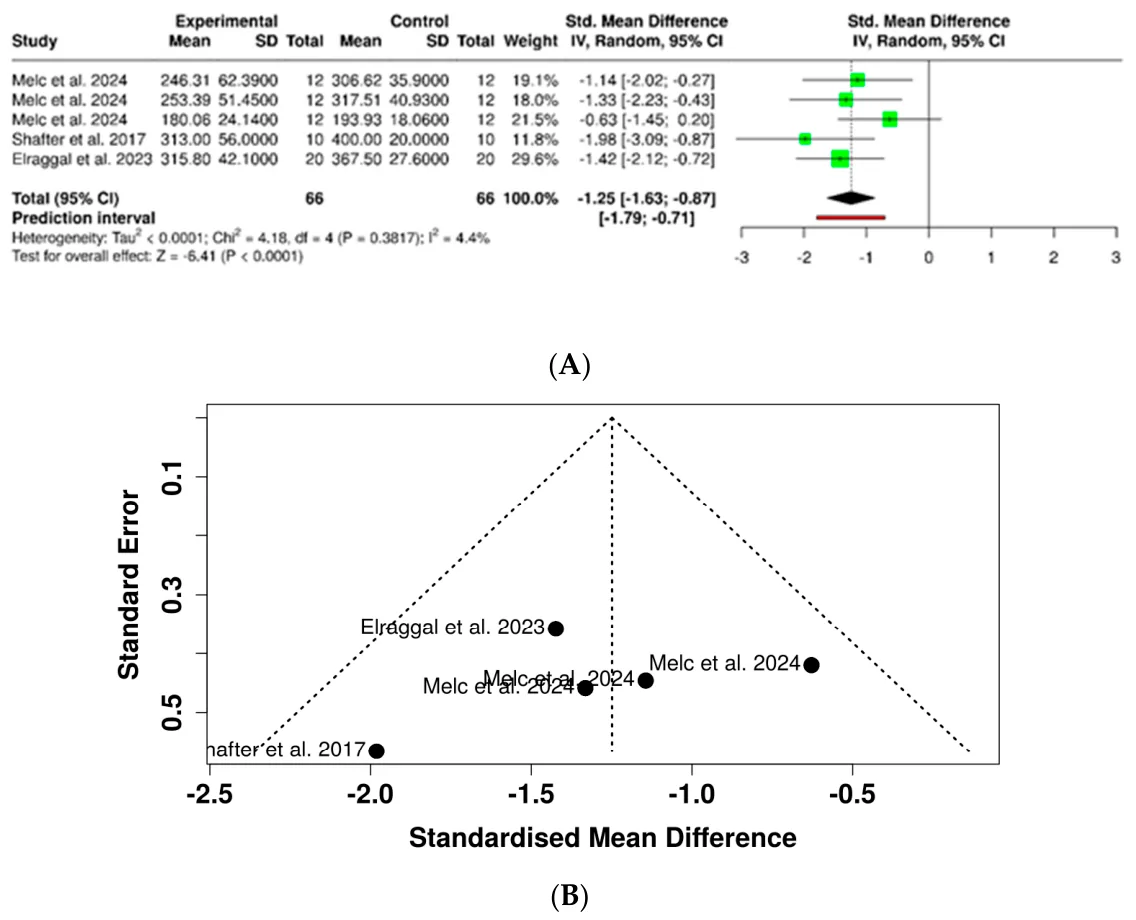
Subgroup analysis of lithium disilicate and lithium silicate CAD/CAM ceramics: effect of artificial aging protocols on flexural strength. (**A**) Forest plot of the random-effects meta-analysis evaluating the effect of artificial aging protocols on the flexural strength of lithium disilicate and lithium silicate CAD/CAM ceramics. (**B**) Funnel plot of the included lithium-based ceramic comparisons [23,25,26].

Five independent comparisons from three studies were included in the lithium-based ceramic subgroup. Artificial aging protocols significantly reduced the flexural strength of lithium disilicate and lithium silicate CAD/CAM ceramics compared with non-aged controls (SMD = −1.25; 95% CI: −1.63 to −0.87; *p* < 0.00001). Low heterogeneity was observed among studies (I^2^ = 4.4%; *p* = 0.382), indicating consistent findings across the included comparisons. The prediction interval remained below the null effect (95% PI: −1.79 to −0.71); nevertheless, its robustness was limited by the inclusion of only three independent studies, and it should therefore be interpreted as exploratory (Figure 4).

### 3.5. Subgroup Analysis: Hybrid and PICN CAD/CAM Ceramics

**Figure 5:**
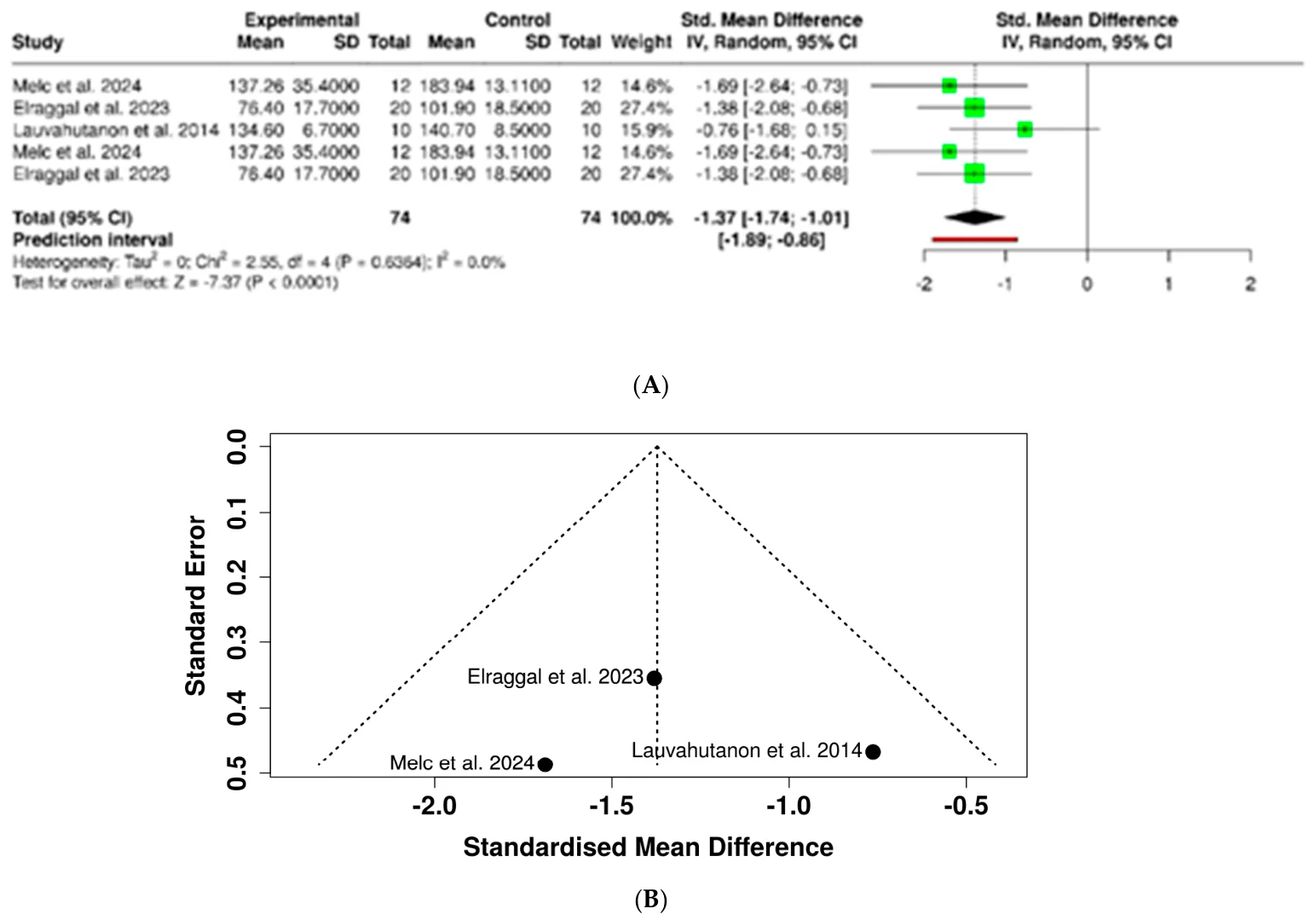
Effect of artificial aging protocols on the flexural strength of hybrid and polymer-infiltrated ceramic network (PICN) CAD/CAM materials. (**A**) Forest plot showing the pooled standardized mean difference (SMD) in flexural strength between aged and non-aged specimens. Negative values indicate reduced flexural strength after aging. (**B**) Funnel plot assessing potential publication bias and small-study effects within the hybrid/PICN subgroup [22,25,26].

Five independent comparisons from three studies were included in the hybrid and polymer-infiltrated ceramic network (PICN) subgroup. Artificial aging protocols significantly reduced the flexural strength of these materials compared with non-aged controls (SMD = −1.37; 95% CI: −1.74 to −1.01; *p* < 0.00001). No significant heterogeneity was observed among the included studies (I^2^ = 0.0%; *p* = 0.636), indicating highly consistent findings. The prediction interval remained below the null effect (95% PI: −1.89 to −0.86); however, because only three independent studies were included, this interval should be considered exploratory and not as definitive evidence of reproducibility (Figure 5).

### 3.6. Subgroup Analysis: Feldspathic and Glass-Based CAD/CAM Ceramics

**Figure 6:**
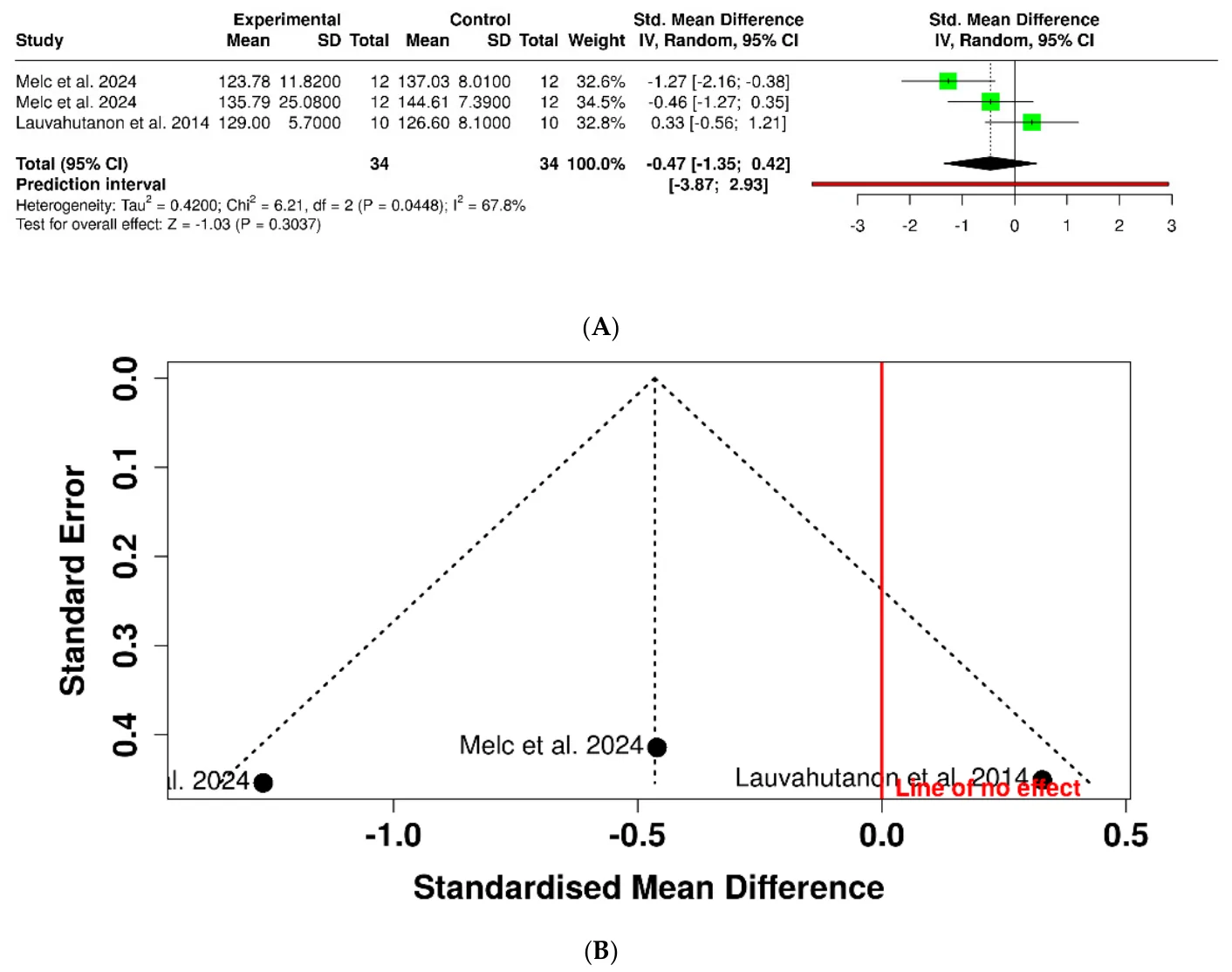
Effect of artificial aging protocols on the flexural strength of feldspathic and glass-based CAD/CAM ceramics. (**A**) Forest plot showing the pooled standardized mean difference (SMD) in flexural strength between aged and non-aged specimens. Negative values indicate reduced flexural strength after aging. (**B**) Funnel plot assessing potential publication bias and small-study effects within the feldspathic and glass-based ceramic subgroup [22,26].

Three independent comparisons from two studies were included in the feldspathic and glass-based ceramic subgroup. Artificial aging protocols did not significantly affect flexural strength compared with non-aged controls (SMD = −0.47; 95% CI: −1.35 to 0.42; *p* = 0.304). Moderate-to-high heterogeneity was observed among the included studies (I^2^ = 67.8%; *p* = 0.045). In addition, the prediction interval was wide (95% PI: −3.87 to 2.93), suggesting substantial variability in the response of these materials to artificial aging protocols. However, because this estimate was based on only two independent studies and three material comparisons, its precision and robustness were limited, and it should be interpreted as exploratory. Overall, the available evidence did not demonstrate a statistically significant reduction in flexural strength following artificial aging protocols (Figure 6).

### 3.7. Sensitivity Analysis

**Figure 7:**
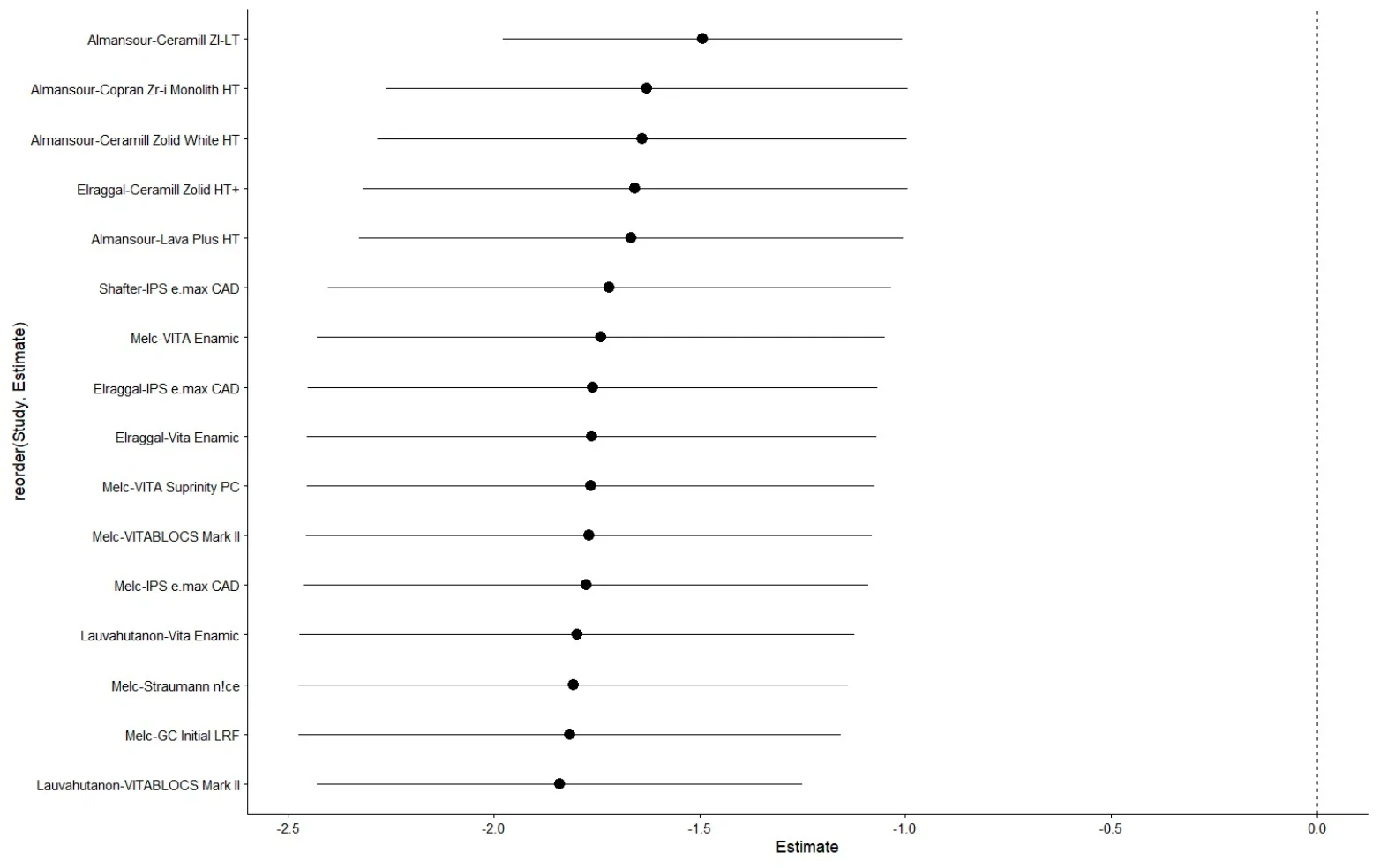
Leave-One-Out Sensitivity Analysis of the Effect of Artificial aging protocols on the Mechanical Properties of CAD/CAM Dental Ceramics.

The leave-one-out sensitivity analysis demonstrated that the overall effect remained stable after the sequential exclusion of each individual comparison (Figure 7). The pooled effect estimates ranged from SMD = −1.49 to −1.84, and all recalculated confidence intervals remained below the null effect. Although the exclusion of the Almansour–Ceramill ZI-LT comparison produced the largest change in the pooled estimate, the direction and statistical significance of the overall effect were preserved. These findings indicate that no single comparison disproportionately influenced the results and support the robustness of the meta-analytic conclusions (Figure 7).

### 3.8. Multilevel Random-Effects Analysis

**Figure 8:**
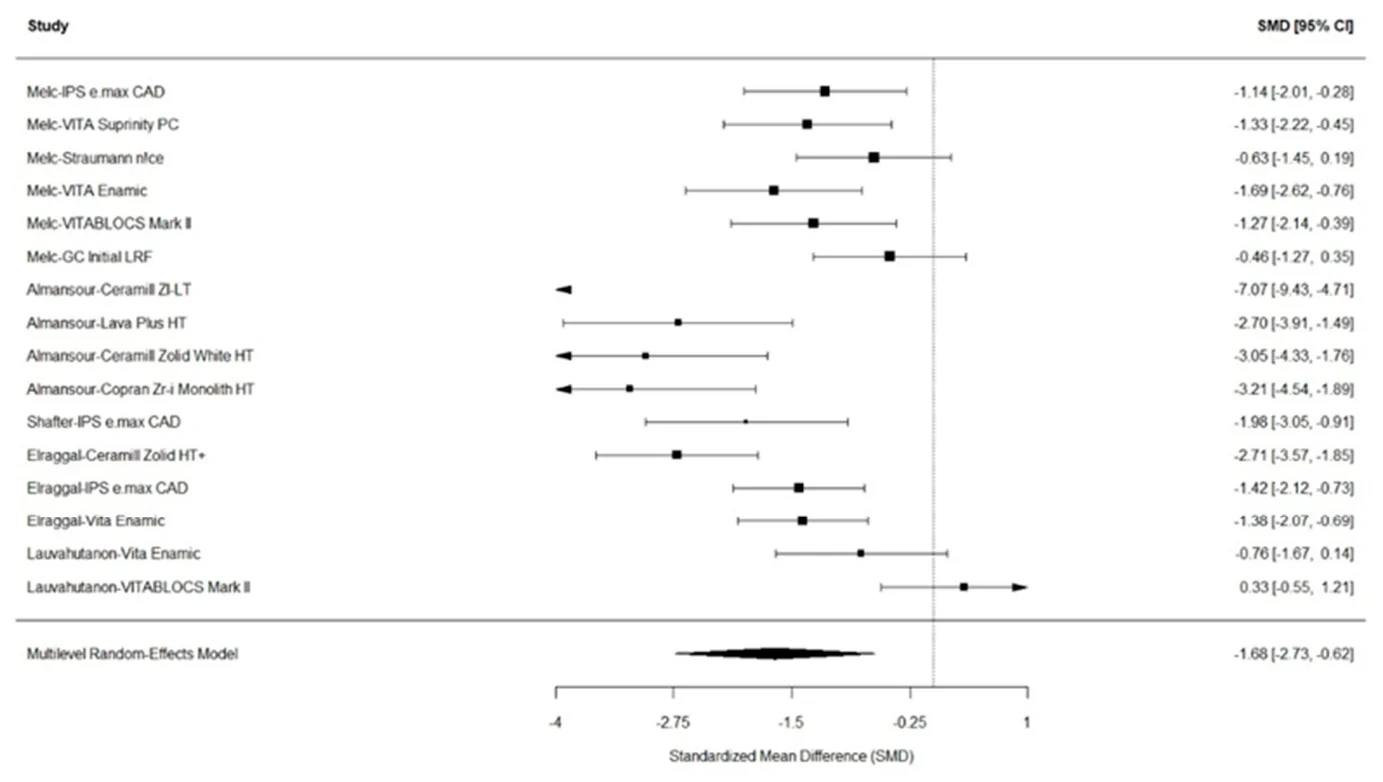
Multilevel random-effects meta-analysis of the effect of artificial aging protocols on the mechanical properties of CAD/CAM dental ceramics.

A multilevel random-effects model was performed to account for the dependency among multiple material comparisons derived from the same study. The analysis confirmed a significant adverse effect of artificial aging protocols on the mechanical properties of CAD/CAM dental ceramics (SMD = −1.68; 95% CI: −2.73 to −0.62). The direction and magnitude of the pooled effect were consistent with those observed in the primary meta-analysis, supporting the robustness of the findings after accounting for the hierarchical structure of the data (Figure 8).

### 3.9. Overall Fracture Resistance

**Figure 9:**
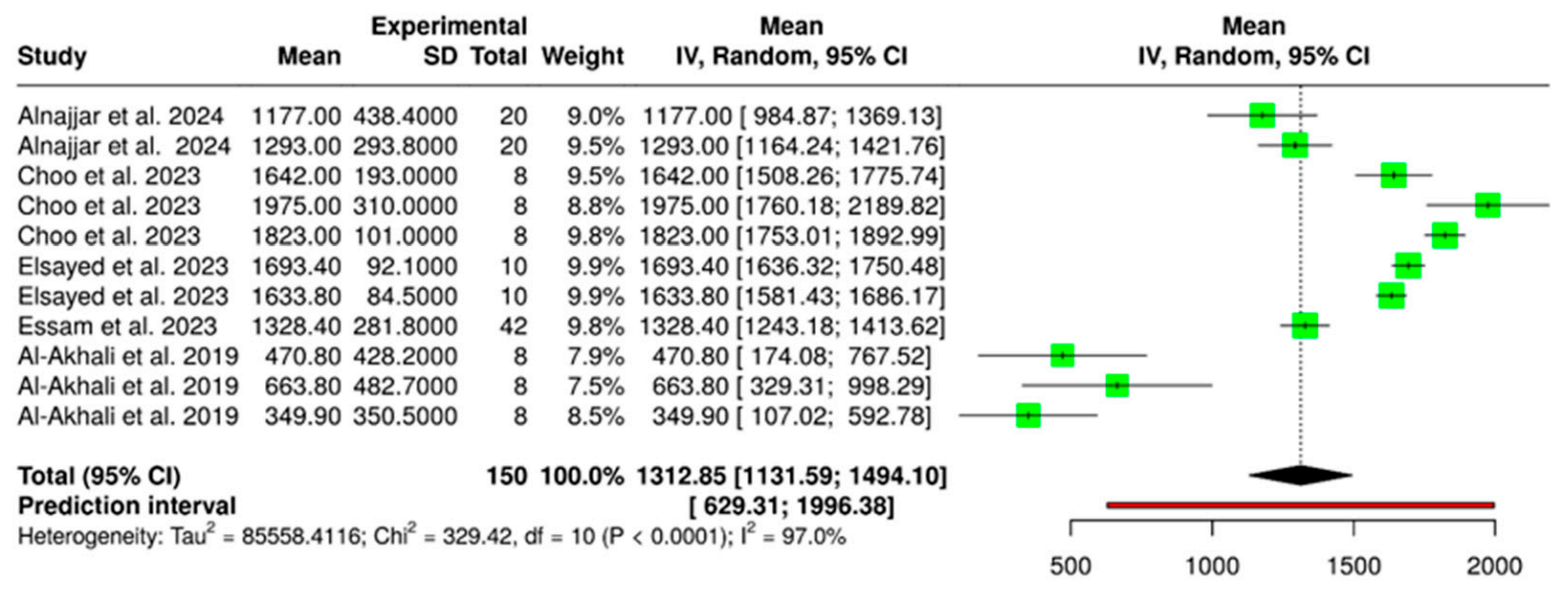
Overall fracture resistance of CAD/CAM dental ceramics after artificial aging protocols [27,28,29,30,31].

A total of 11 comparisons from five studies were included in the quantitative synthesis of fracture resistance. The random-effects model yielded a pooled fracture resistance of 1312.85 N (95% CI: 1131.59–1494.10 N) following thermocycling and artificial aging protocols (Figure 9). Considerable heterogeneity was observed among the included comparisons (I^2^ = 97.0%; *p* < 0.0001), reflecting substantial variability in ceramic composition, restoration design, and aging protocols. The prediction interval was wide (629.31–1996.38 N), indicating that fracture resistance values may vary considerably across different CAD/CAM ceramic materials and experimental conditions.

### 3.10. Sensitivity Analysis

**Figure 10:**
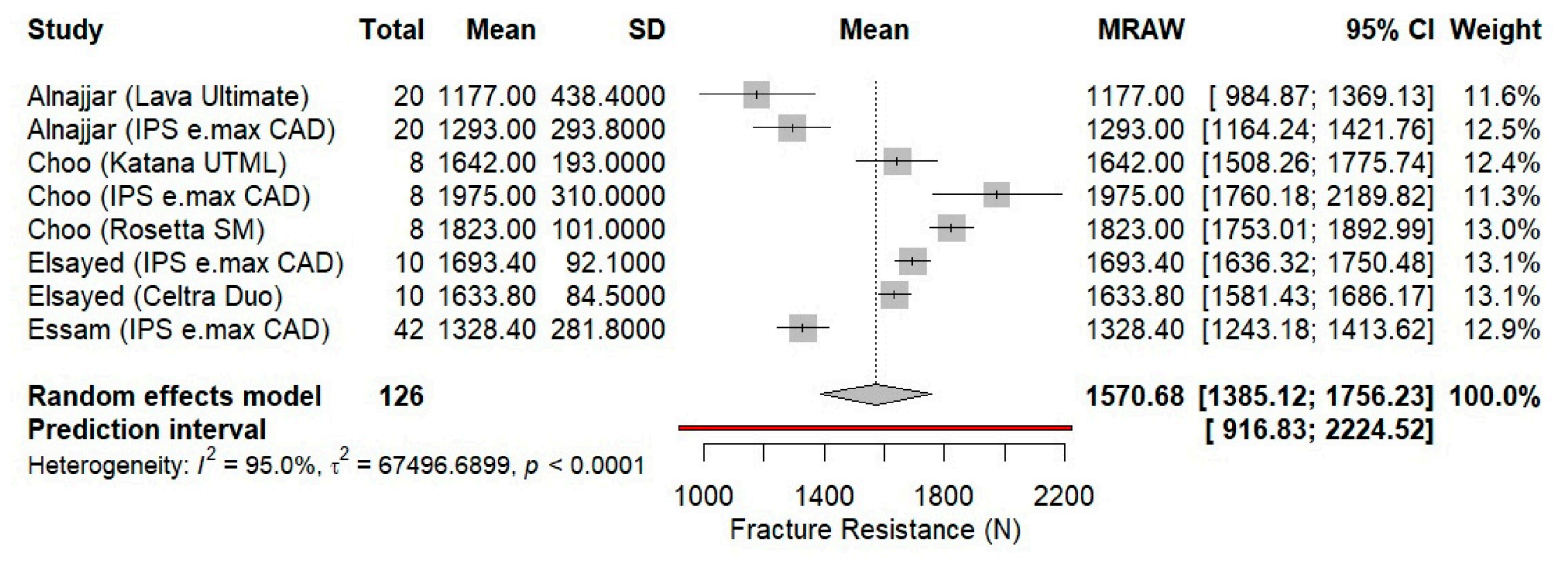
Sensitivity Analysis of Pooled Fracture Resistance Following Exclusion of Al-Akhali et al. (2019) [27].

A sensitivity analysis was performed by excluding the study conducted by Al-Akhali et al. (2019) [27], which reported the lowest fracture resistance values among the included comparisons (Figure 10). Following exclusion, the pooled fracture resistance increased to 1570.68 N (95% CI: 1385.12–1756.23 N). Although heterogeneity remained substantial (I^2^ = 95.0%; *p* < 0.0001), the overall findings were consistent with the primary analysis, indicating that CAD/CAM ceramics maintained high fracture resistance after artificial aging protocols. These results suggest that the overall estimate was influenced by the lower values reported by Al-Akhali et al., but the general conclusions remained unchanged.

### 3.11. Subgroup Analysis: Lithium Disilicate Ceramics (IPS e.max CAD)

**Figure 11:**
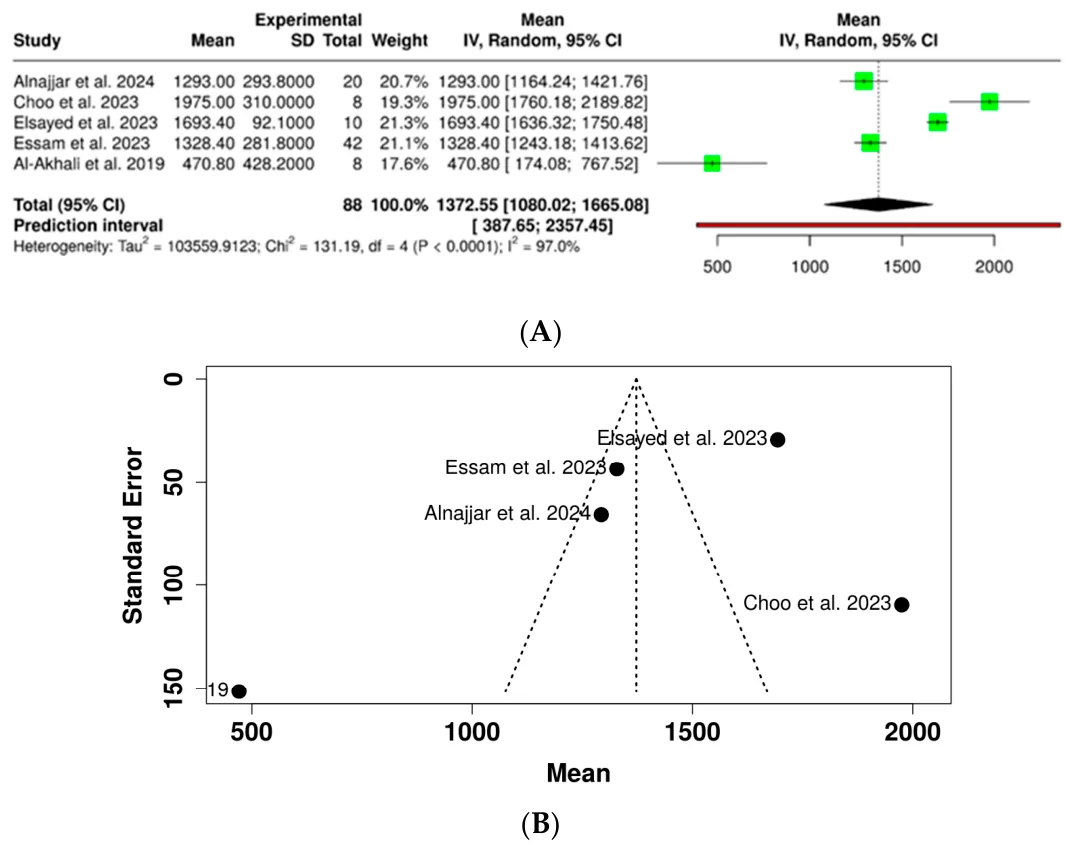
Fracture resistance of lithium disilicate CAD/CAM ceramics (IPS e.max CAD) after artificial aging protocols. (**A**) Forest plot showing the pooled mean fracture resistance (N) of IPS e.max CAD restorations subjected to artificial aging protocols. (**B**) Funnel plot assessing potential publication bias and small-study effects within the lithium disilicate subgroup [27,28,29,30,31].

Five comparisons evaluating IPS e.max CAD restorations were included in the lithium disilicate subgroup analysis. The pooled fracture resistance after artificial aging protocols was 1372.55 N (95% CI: 1080.02–1665.08 N) (Figure 11). Substantial heterogeneity was observed among the included studies (I^2^ = 97.0%; *p* < 0.0001). The prediction interval was wide (387.65–2357.45 N), indicating considerable variability in fracture resistance values across experimental conditions. Despite this heterogeneity, lithium disilicate ceramics demonstrated high fracture resistance following artificial aging protocols.

### 3.12. Subgroup Analysis: Zirconia and Zirconia-Reinforced Lithium Silicate (ZLS) Ceramics

**Figure 12:**
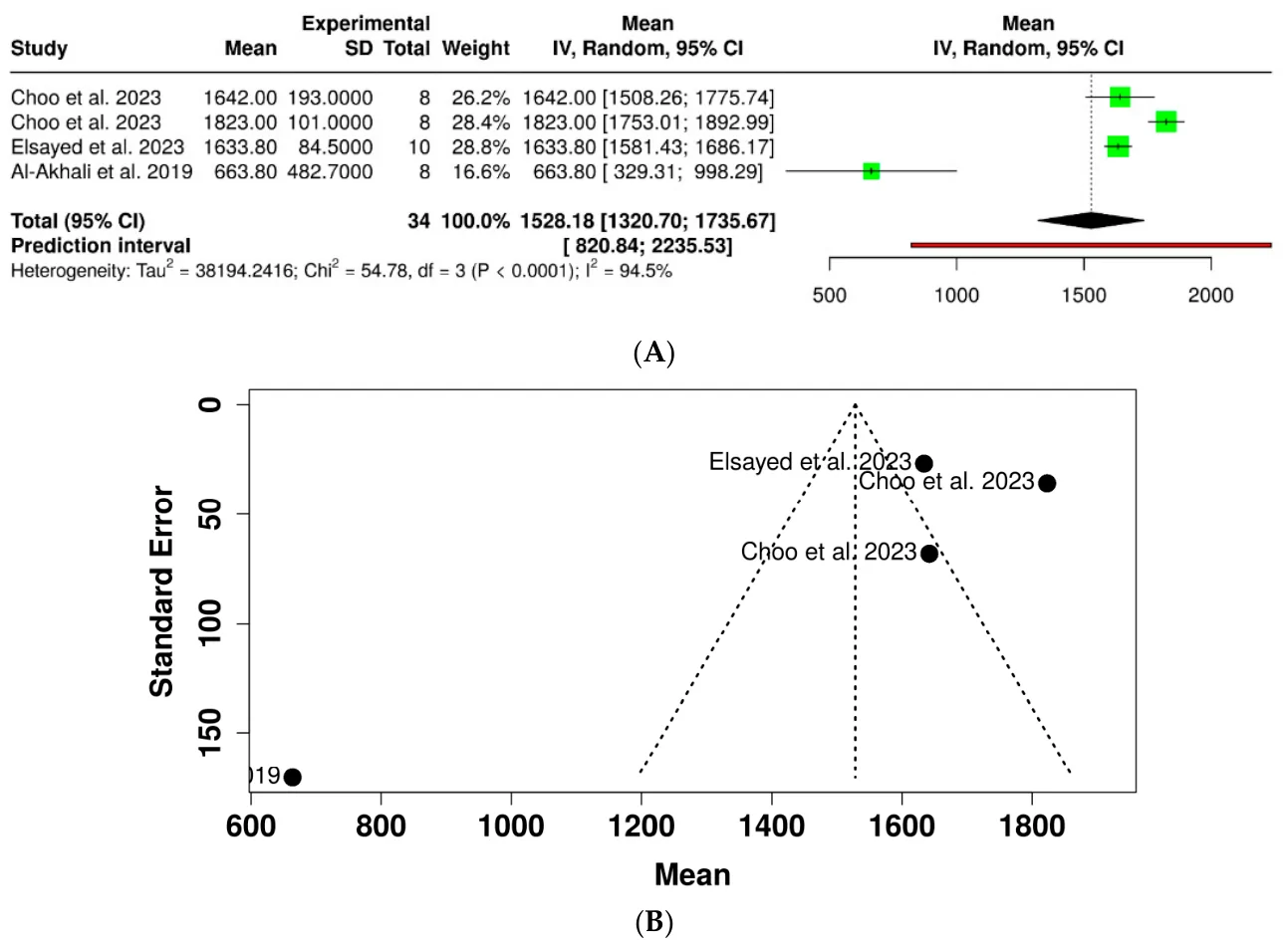
Fracture resistance of zirconia and zirconia-reinforced lithium silicate (ZLS) CAD/CAM ceramics after artificial aging protocols. (**A**) Forest plot showing the pooled mean fracture resistance (N) of zirconia and ZLS materials subjected to artificial aging protocols. (**B**) Funnel plot assessing potential publication bias and small-study effects within the zirconia/ZLS subgroup [27,28,29].

Four comparisons evaluating zirconia and zirconia-reinforced lithium silicate (ZLS) ceramics were included in this subgroup analysis. The pooled fracture resistance after artificial aging protocols was 1528.18 N (95% CI: 1320.70–1735.67 N) (Figure 12). Considerable heterogeneity was detected among the included studies (I^2^ = 94.5%; *p* < 0.0001). The prediction interval ranged from 820.84 to 2235.53 N, indicating substantial variability in fracture resistance across different zirconia-based and ZLS materials. Overall, these materials exhibited high fracture resistance following artificial aging protocols.

### 3.13. Subgroup Analysis: Hybrid Ceramics, PICN, and Resin Nanoceramics

**Figure 13:**
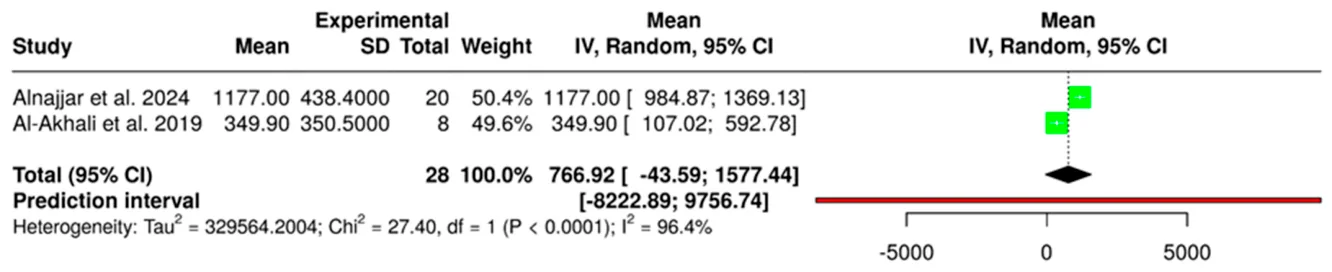
Fracture resistance of hybrid ceramics, polymer-infiltrated ceramic network (PICN), and resin nanoceramic CAD/CAM materials after artificial aging protocols [27,31].

Two comparisons evaluating hybrid ceramics, polymer-infiltrated ceramic network (PICN), and resin nanoceramic materials were included in this subgroup analysis. The pooled fracture resistance after artificial aging protocols was 766.92 N (95% CI: −43.59 to 1577.44 N) (Figure 13). Substantial heterogeneity was observed between studies (I^2^ = 96.4%; *p* < 0.0001), and the prediction interval was extremely wide (−8222.89 to 9756.74 N), indicating considerable uncertainty in the pooled estimate. Consequently, no statistically significant pooled effect could be established for this subgroup.

### 3.14. Sensitivity Analysis

**Figure 14:**
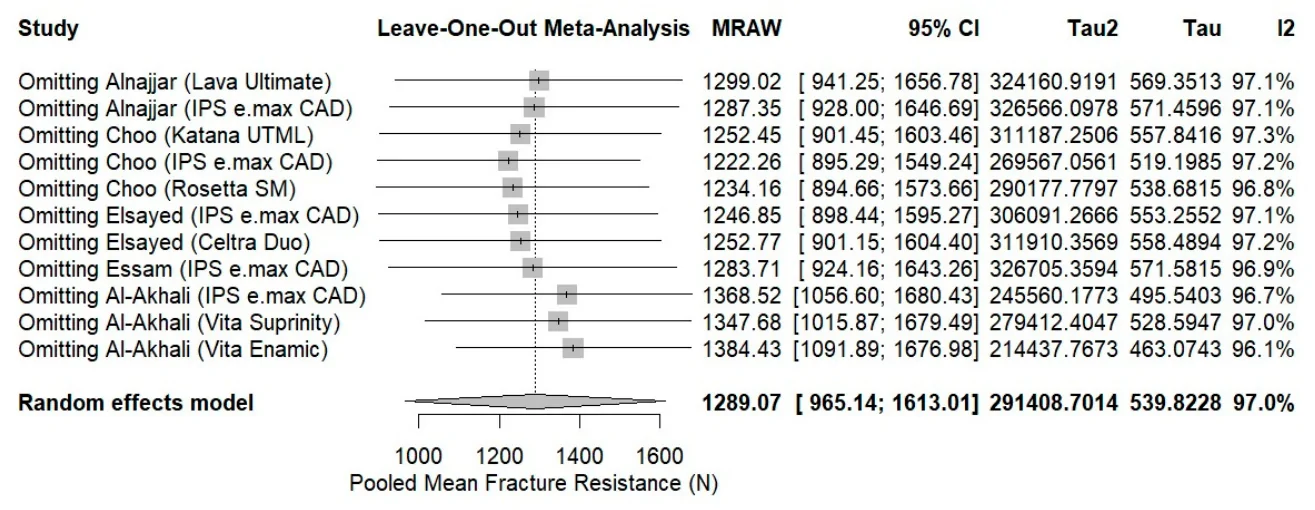
Leave-one-out sensitivity analysis of fracture resistance in CAD/CAM dental ceramics after artificial aging protocols.

The leave-one-out sensitivity analysis demonstrated that the pooled fracture resistance remained stable after the sequential exclusion of each individual comparison (Figure 14). The pooled estimates ranged from 1222.26 N to 1384.43 N, with all recalculated confidence intervals overlapping the overall pooled estimate. Heterogeneity remained consistently high across analyses (I^2^ = 96.1–97.3%), indicating that no single comparison was primarily responsible for the observed between-study variability. These findings support the robustness of the pooled fracture resistance estimate and suggest that the overall results were not disproportionately influenced by any individual study or material.

## 4. Discussion

The present systematic review and meta-analysis demonstrated that artificial aging protocols significantly reduce the flexural strength of CAD/CAM dental ceramics (SMD = −1.68; 95% CI: −2.28 to −1.08; *p* < 0.00001). Although substantial heterogeneity was observed (I^2^ = 78.5%), the direction of the effect remained consistent across studies, indicating that artificial aging protocols exert a detrimental influence on the mechanical integrity of contemporary ceramic materials. These findings support the concept that prolonged exposure to moisture, thermal fluctuations, and cyclic loading promotes structural degradation and compromises the long-term mechanical performance of CAD/CAM restorations [24,25,26,32,33,34,35,36,37,38,39,40,41].

Among all evaluated ceramic categories, zirconia-based materials exhibited the greatest reduction in flexural strength after aging (SMD = −3.31; 95% CI: −4.27 to −2.35; *p* < 0.00001). This finding is biologically plausible because zirconia is susceptible to low-temperature degradation (LTD), a hydrothermal phenomenon characterized by tetragonal-to-monoclinic phase transformation, surface roughening, grain pullout, and microcrack formation [6,8,10,32,33,34,35,36]. Lughi and Sergo [32] described LTD as one of the main aging-related limitations of zirconia in dentistry, while Harada et al. [33] reported that accelerated aging may reduce fracture toughness. Inokoshi et al. [34] demonstrated that sintering conditions influence low-temperature degradation, and Yang et al. [36] confirmed through systematic review and meta-analysis that LTD significantly affects the surface characteristics of Y-TZP ceramics. More recently, Zhai et al. [35] reported that low-temperature degradation remains relevant even for zirconia ceramics fabricated using stereolithography. Likewise, the studies included in the present review showed significant reductions in strength after thermomechanical aging and fatigue loading [24,25]. Although zirconia remains one of the strongest restorative ceramics available, these findings indicate that its mechanical properties may progressively deteriorate under prolonged aging conditions.

Lithium disilicate and lithium silicate ceramics also showed a significant reduction in flexural strength (SMD = −1.25; 95% CI: −1.63 to −0.87; *p* < 0.00001), although the magnitude of degradation was substantially lower than that observed for zirconia. The low heterogeneity detected within this subgroup (I^2^ = 4.4%) suggests a predictable aging response. The relatively favorable performance of lithium disilicate is likely related to its interlocking crystalline microstructure, which promotes crack deflection and energy dissipation during loading [13,20,37,38,39]. Nevertheless, repeated thermocycling and moisture exposure may weaken the glassy matrix and facilitate subcritical crack propagation. Nawafleh et al. [37] emphasized that fatigue testing parameters significantly influence the interpretation of lithium disilicate restoration performance. In addition, Rekow et al. [40] and Kelly [41] highlighted that ceramic fracture behavior is strongly related to flaw distribution, crack propagation, and testing configuration. The studies by Shafter et al. [23], Elraggal et al. [25], and Melc et al. [26] are consistent with this interpretation, as they demonstrated reductions in flexural strength after aging. From a clinical perspective, these results reinforce the long-term reliability of lithium disilicate restorations despite measurable reductions in mechanical properties after artificial aging.

Hybrid ceramics and polymer-infiltrated ceramic network (PICN) materials demonstrated a significant reduction in flexural strength (SMD = −1.37; 95% CI: −1.74 to −1.01; *p* < 0.00001) with no detectable heterogeneity (I^2^ = 0.0%). Their aging behavior appears closely related to the presence of a polymer network infiltrating a ceramic scaffold. Although this structure improves stress distribution and reduces brittleness, it also increases susceptibility to hydrolytic degradation and water sorption. Water uptake may plasticize the polymer phase, weaken the ceramic-polymer interface, and accelerate crack formation. Similar mechanisms have been reported by Facenda et al. [42], Della Bona et al. [43], He and Swain [44], Coldea et al. [45], and El Zhawi et al. [46], who described the structure, mechanical behavior, and fatigue resistance of PICN materials. Furthermore, Mainjot et al. [38] and Alamoush et al. [47] emphasized that CAD/CAM composite-based materials remain particularly susceptible to hydrolytic degradation because of their polymeric components. Therefore, while hybrid ceramics provide biomechanical advantages because of their dentin-like elastic modulus, their long-term performance may be influenced by environmental degradation.

In contrast, feldspathic and glass-based CAD/CAM ceramics did not demonstrate a statistically significant reduction in flexural strength after aging (SMD = −0.47; 95% CI: −1.35 to 0.42; *p* = 0.304). However, this finding should be interpreted cautiously because only a limited number of comparisons were available and heterogeneity remained substantial (I^2^ = 67.8%). Differences in microstructure, crystalline content, flaw distribution, elastic properties, and testing methodologies may partially explain the observed variability. Belli et al. [39] demonstrated that chairside CAD/CAM materials differ considerably in elastic constants and microstructural organization, Spitznagel et al. [12] highlighted material-dependent differences among CAD/CAM ceramic restorative systems. Yılmaz et al. [48] further showed that hydrothermal aging may affect the flexural strength and microhardness of materials used for additive or subtractive manufacturing of definitive restorations. Consequently, the current evidence remains insufficient to establish definitive conclusions regarding the aging behavior of contemporary feldspathic CAD/CAM ceramics.

Although aging significantly affected flexural strength, fracture resistance remained comparatively stable. The pooled fracture resistance after aging reached 1312.85 N (95% CI: 1131.59–1494.10 N), indicating that most CAD/CAM ceramics retained substantial load-bearing capacity despite prolonged thermocycling and fatigue. Studies included in the quantitative synthesis consistently demonstrated fracture resistance values exceeding physiological masticatory loads [27,28,29,30,31]. Similar observations have been reported in studies evaluating the mechanical reliability and fatigue behavior of CAD/CAM restorations. Fouda et al. [49] investigated fatigue, fracture resistance, and color properties of esthetic CAD/CAM monolithic ceramics, while Rekow et al. [40], Kelly [41], and Guess et al. [50] emphasized that ceramic reliability depends not only on intrinsic material strength but also on defect population, restoration geometry, surface condition, and loading configuration. This distinction likely explains why reductions in flexural strength do not necessarily translate into clinically relevant decreases in fracture resistance.

The robustness of the present findings was confirmed through sensitivity analyses and multilevel random-effects modeling. Sequential exclusion of individual studies produced only minor variations in effect size, and all recalculated estimates remained statistically significant. Likewise, the multilevel model yielded results nearly identical to those obtained in the primary analysis, indicating that no individual comparison disproportionately influenced the overall conclusions. These observations strengthen confidence in the reliability of the pooled estimates and support the consistency of the observed aging effect.

The substantial heterogeneity observed in the overall analysis is likely explained by differences in ceramic composition, aging protocols, storage conditions, fatigue regimens, specimen geometry, and testing methodologies. The included studies employed artificial aging protocols ranging from a few thousand thermocycles to more than one million fatigue cycles, representing markedly different levels of simulated clinical service. Although the prediction intervals for zirconia, lithium disilicate/lithium silicate, and hybrid/PICN materials remained below the null effect, these estimates were based on only two or three independent studies per subgroup. Therefore, they should be regarded as exploratory and should not be interpreted as precise predictions of the effects expected in future studies. Nevertheless, the direction of the pooled estimates generally suggested a detrimental influence of aging on flexural strength. This cautious interpretation is consistent with previous evidence showing that the mechanical response of CAD/CAM ceramics is strongly influenced by microstructure, manufacturing route, and test configuration [38,39,40,41,46,47,48,49,50].

This review possesses several important strengths. To our knowledge, it is the first quantitative synthesis specifically evaluating the effects of artificial aging protocols on CAD/CAM dental ceramics across multiple material categories. The use of subgroup analyses, multilevel modeling, prediction intervals, sensitivity analyses, and risk-of-bias assessment provides a comprehensive evaluation of the available evidence [21]. Furthermore, the inclusion of zirconia, lithium disilicate, feldspathic ceramics, glass ceramics, and PICN materials offers a broad perspective on how different microstructures respond to aging.

Nevertheless, several limitations should be acknowledged. All included investigations were conducted in vitro and therefore cannot fully reproduce the complexity of the oral environment. In addition, substantial variability existed among aging protocols, specimen geometries, restoration designs, and mechanical testing methods. The limited number of studies available for some subgroup analyses, particularly those involving feldspathic and glass-based ceramics, may also have reduced statistical power. The small number of independent studies and comparisons also limited the robustness of the prediction intervals and the reliability of funnel-plot interpretation. Consequently, these analyses should be regarded as exploratory, and publication bias or small-study effects cannot be reliably confirmed or excluded. Finally, considerable diversity exists within contemporary CAD/CAM material systems, and materials classified within the same subgroup may differ substantially in composition, translucency, crystalline structure, polymer content, and manufacturing procedures.

Although standardized mean differences allowed the relative effect of aging to be expressed on a common dimensionless scale, they do not make three-point bending and biaxial flexural-strength tests physically equivalent. Differences in specimen geometry, stress distribution, effective testing area, flaw exposure, and failure initiation may have contributed to the observed heterogeneity. Because only one included study used biaxial flexural-strength testing, a reliable subgroup analysis according to testing method could not be performed. Therefore, the magnitude of the pooled flexural-strength estimate should be interpreted cautiously.

An additional limitation of this review is that direct compressive strength was not included among the prespecified outcomes, and cyclic compressive fatigue was not quantitatively synthesized as a separate outcome. These mechanical properties may be particularly relevant for minimally invasive restorations, such as thin occlusal veneers, in which restoration thickness, adhesive support, supporting substrate, and stress distribution may influence failure behavior. Although some included studies evaluated mechanical fatigue and load-to-fracture performance under occlusal-type loading, these outcomes should not be considered equivalent to direct compressive strength testing.

The fracture-resistance synthesis should be interpreted differently from the flexural-strength meta-analysis. Whereas the flexural-strength analysis quantified the difference between aged and non-aged specimens, the fracture-resistance analysis pooled absolute post-aging values because comparable non-aged controls were not consistently available. Therefore, the pooled fracture-resistance estimate describes the performance of restorations after the aging protocols evaluated but does not quantify the detrimental effect attributable to aging. Direct comparisons between these two pooled analyses are not appropriate.

Another important limitation is that the quantitative synthesis did not specifically evaluate chemical degradation produced by artificial saliva, acidic media, or repeated pH fluctuations. Furthermore, the available evidence did not permit assessment of the interaction between erosive chemical challenges and cyclic occlusal or compressive loading. Because CAD/CAM restorations are continuously exposed to both chemical and mechanical challenges in the oral environment, the clinical applicability of the present findings should be interpreted with caution. The pooled estimates primarily reflect the effects of the thermal, hydrothermal, and mechanical aging protocols evaluated in the included studies and should not be directly extrapolated to the combined erosive–mechanical conditions present in the oral environment. This limitation is particularly relevant to minimally invasive restorations, such as thin occlusal veneers, whose clinical behavior may depend on the simultaneous effects of restoration thickness, adhesive support, chemical degradation, and cyclic occlusal loading.

The substantial heterogeneity observed in the analyses may be partly explained by the marked variability in artificial aging protocols. The included protocols differed in both mechanism and intensity, ranging from isolated water storage or thermocycling to combined thermomechanical aging involving 3500–10,000 thermocycles and 100,000–1,200,000 fatigue cycles. Consequently, the pooled estimates should be interpreted as average effects across heterogeneous laboratory aging conditions and not as the effect of a single standardized or clinically equivalent aging protocol. Detailed protocol characteristics are summarized in Table 2 and Table 4.

From a clinical perspective, the present findings indicate that material selection should consider not only baseline mechanical properties but also long-term aging behavior. Within the included flexural-strength comparisons, zirconia-based ceramics exhibited the greatest pooled reduction after aging, whereas lithium disilicate and lithium silicate ceramics demonstrated a more consistent aging response. Hybrid and PICN materials showed favorable biomechanical characteristics but may remain susceptible to hydrolytic degradation because of their polymer-containing structure. Nevertheless, these laboratory findings should not be considered direct predictors of clinical survival. Long-term durability should therefore be considered alongside esthetics, restoration design, material thickness, adhesive support, and immediate mechanical performance when selecting CAD/CAM restorative materials.

Future research should focus on the development of standardized multifactorial aging protocols and on the evaluation of emerging CAD/CAM materials, including ultra-translucent zirconias, advanced lithium silicate ceramics, next-generation hybrid systems, and novel polymer-infiltrated ceramic networks. A specific priority should be a standardized 2 × 2 factorial in vitro study in which contemporary CAD/CAM ceramics are allocated to four conditions: untreated control, acidic exposure or pH cycling alone, cyclic compressive loading alone, and combined acidic exposure with cyclic compressive loading. This experimental design would allow the independent effects of chemical erosion and mechanical fatigue, as well as their potential synergistic interaction, to be quantified. Outcomes should include flexural strength, fracture resistance, fatigue survival, surface roughness, microhardness, microstructural changes, and failure patterns. Particular attention should be given to minimally invasive restorations, such as thin occlusal veneers, in which restoration thickness, adhesive support, and supporting substrate may modify the erosive–mechanical response. Long-term laboratory and clinical investigations are required to determine whether these chemical and mechanical challenges exert independent, additive, or synergistic effects and whether laboratory reductions in mechanical properties translate into clinically meaningful differences in restoration survival. Future systematic reviews should specifically evaluate studies using combined erosive–mechanical aging protocols when a sufficient body of evidence becomes available.

Overall, the present systematic review and meta-analysis demonstrated that thermocycling and the artificial aging protocols evaluated in the included studies significantly compromised the flexural strength of CAD/CAM dental ceramics. Zirconia-based materials exhibited the greatest pooled reduction, followed by hybrid/PICN and lithium disilicate/lithium silicate ceramics, whereas the evidence regarding feldspathic and glass-based ceramics remained inconclusive. Although aged CAD/CAM restorations generally retained substantial load-to-fracture values, these findings should not be interpreted as demonstrating universal clinical acceptability because restoration design, material thickness, supporting substrate, and clinical loading conditions varied across studies. Material composition appears to be an important determinant of aging susceptibility, highlighting the need to consider long-term degradation behavior when selecting CAD/CAM restorative materials.

## 5. Conclusions

Artificial aging protocols significantly reduced the flexural strength of CAD/CAM dental ceramics, confirming that artificial aging protocols negatively affect their mechanical performance. Zirconia-based ceramics exhibited the greatest susceptibility to degradation, followed by lithium disilicate and hybrid/PICN materials, whereas evidence for feldspathic ceramics remains limited and inconclusive. Despite these reductions, fracture resistance generally remained within clinically acceptable ranges after aging. Material composition appears to be the primary determinant of aging susceptibility, highlighting the importance of considering long-term degradation behavior when selecting CAD/CAM restorative materials. Further standardized in vitro and clinical studies are required to better understand the long-term impact of aging on restoration longevity.

## Figures and Tables

**Table 1:** Characteristics of the included studies.

Study	Year	Ceramic Category	CAD/CAM Material	n per Group	Outcomes Evaluated	Aging Protocol	Main Direction of Results
Lauvahutanon et al. [22]	2014	Hybrid/PICN, Feldspathic Ceramic	VITA Enamic; VITABLOCS Mark II	10	Flexural strength	Water storage + 10,000 thermocycles (5–55 °C)	Minimal effect on flexural strength; material-dependent response
Shafter et al. [23]	2017	Lithium Disilicate	IPS e.max CAD	10	Flexural strength	5000 thermocycles (5–55 °C)	Aging reduced flexural strength
Almansour et al. [24]	2018	Zirconia	Ceramill ZI-LT; Lava Plus HT; Ceramill Zolid White HT; Copran Zr-i Monolith HT	10	Biaxial flexural strength	3500 thermocycles + 250,000 fatigue cycles	Significant reduction in flexural strength after aging
Elraggal et al. [25]	2023	Zirconia; Lithium Disilicate; PICN	Ceramill Zolid HT+; IPS e.max CAD; VITA Enamic	20	Flexural strength	1,000,000 fatigue cycles	Aging significantly decreased flexural strength
Melc et al. [26]	2024	Lithium Disilicate; Lithium Silicate; PICN; Feldspathic; Glass Ceramic	IPS e.max CAD; VITA Suprinity PC; Straumann n!ce; VITA Enamic; VITABLOCS Mark II; GC Initial LRF	12	Flexural strength	Thermomechanical aging (100,000 cycles)	Material-dependent reduction in flexural strength

**Table 2:** Detailed methodological and material characteristics of the included studies.

Study	Ceramic Type	CAD/CAM Material	Aging Method	Thermocycling	Mechanical Fatigue	Number of Cycles	Flexural Test	Standard/ Conditions
Lauvahutanon et al. [22]	Hybrid/PICN; Feldspathic	VITA Enamic; VITABLOCS Mark II	Water storage + thermocycling	Yes	No	10,000 thermocycles	Three-point bending	ISO 6872 [22]
Shafter et al. [23]	Lithium Disilicate	IPS e.max CAD	Thermocycling	Yes	No	5000 thermocycles	Three-point bending	Universal testing machine
Almansour et al. [24]	Zirconia	Ceramill ZI-LT; Lava Plus HT; Ceramill Zolid White HT; Copran Zr-i Monolith HT	Thermomechanical aging	Yes	Yes	3500 thermocycles + 250,000 fatigue cycles	Biaxial flexural strength	ISO 6872
Elraggal et al. [25]	Zirconia; Lithium Disilicate; PICN	Ceramill Zolid HT+; IPS e.max CAD; VITA Enamic	Mechanical fatigue	No	Yes	1,000,000 cycles	Three-point bending	Water storage at 37 °C
Melc et al. [26]	Lithium Disilicate; Lithium Silicate; PICN; Feldspathic; Glass Ceramic	Multiple CAD/CAM ceramic materials	Thermomechanical aging	Yes	Yes	100,000 cycles	Three-point bending	Standardized laboratory conditions

**Table 3:** Characteristics of the studies included in the quantitative synthesis of fracture resistance.

Study	Year	Ceramic Category	CAD/CAM Material	n per Group	Outcome Evaluated	Aging Protocol	Main Direction of Results
Al-Akhali et al. [27]	2019	Lithium Disilicate; Zirconia-Reinforced Lithium Silicate; PICN	IPS e.max CAD; VITA Suprinity; VITA Enamic	8	Fracture resistance	Thermomechanical aging (1,200,000 cycles + 5500 thermocycles)	Fracture resistance varied according to ceramic composition; VITA Suprinity showed the highest resistance among aged restorations
Choo et al. [28]	2023	Zirconia; Lithium Disilicate; Zirconia-Reinforced Lithium Silicate	Katana UTML; IPS e.max CAD; Rosetta SM	8	Fracture resistance	10,000 thermocycles + 200,000 fatigue cycles	IPS e.max CAD demonstrated the highest fracture resistance after aging
Elsayed et al. [29]	2023	Lithium Disilicate; Zirconia-Reinforced Lithium Silicate	IPS e.max CAD; Celtra Duo	10	Fracture resistance	Mechanical fatigue (120,000 cycles, 100 N)	Both materials maintained high fracture resistance after artificial aging
Essam et al. [30]	2023	Lithium Disilicate	IPS e.max CAD	42	Fracture resistance	Water storage (75 days) + 240,000 fatigue cycles	Fracture resistance was influenced by restoration thickness and surface treatment
Alnajjar et al. [31]	2024	Lithium Disilicate; Resin Nanoceramic	IPS e.max CAD; Lava Ultimate	20	Fracture resistance	Thermocycling + mechanical fatigue	IPS e.max CAD exhibited greater fracture resistance than Lava Ultimate after aging

**Table 4:** Detailed methodological and material characteristics of the studies included in the fracture resistance meta-analysis.

Study	Ceramic Type	CAD/CAM Material	Aging Method	Thermocycling	Mechanical Fatigue	Number of Cycles	Fracture Test	Standard/ Conditions
Al-Akhali et al. [27]	Lithium Disilicate; ZLS; PICN	IPS e.max CAD; VITA Suprinity; VITA Enamic	Thermomechanical aging	Yes	Yes	5500 thermocycles + 1,200,000 cycles	Load-to-fracture test	98 N, 2.4 Hz
Choo et al. [28]	Zirconia; Lithium Disilicate; ZLS	Katana UTML; IPS e.max CAD; Rosetta SM	Thermomechanical aging	Yes	Yes	10,000 thermocycles + 200,000 cycles	Load-to-fracture test	70 N, 1 Hz
Elsayed et al. [29]	Lithium Disilicate; ZLS	IPS e.max CAD; Celtra Duo	Mechanical fatigue	No	Yes	120,000 cycles	Load-to-fracture test	100 N
Essam et al. [30]	Lithium Disilicate	IPS e.max CAD	Water storage + fatigue aging	No	Yes	240,000 cycles	Load-to-fracture test	49 N, 1.7 Hz
Alnajjar et al. [31]	Lithium Disilicate; Resin Nanoceramic	IPS e.max CAD; Lava Ultimate	Thermocycling + fatigue aging	Yes	Yes	2500 thermocycles + 250,000 cycles	Load-to-fracture test	50 N, 1.25 Hz

**Table 5:** Risk of Bias Assessment of Included Studies Using the RoB-Iv Tool.

Study	Specimen Preparation	Randomization	Blinding	Outcome Measurement	Statistical Analysis	Overall Risk
Lauvahutanon et al. (2014) [22]	Low	Some Concerns	Some Concerns	Low	Low	Some Concerns
Shafter et al. (2017) [23]	Low	Some Concerns	Some Concerns	Low	Low	Some Concerns
Almansour et al. (2018) [24]	Low	Low	Some Concerns	Low	Low	Low
Elraggal et al. (2023) [25]	Low	Some Concerns	Some Concerns	Low	Low	Some Concerns
Melc et al. (2024) [26]	Low	Low	Some Concerns	Low	Low	Low
Al-Akhali et al. (2019) [27]	Low	Some Concerns	Some Concerns	Low	Low	Some Concerns
Choo et al. (2023) [28]	Low	Low	Some Concerns	Low	Low	Low
Elsayed et al. (2023) [29]	Low	Some Concerns	Some Concerns	Low	Low	Some Concerns
Essam et al. (2023) [30]	Low	Low	Some Concerns	Low	Low	Low
Alnajjar et al. (2024) [31]	Low	Some Concerns	Some Concerns	Low	Low	Some Concerns

**Table 6:** Summary of Risk of Bias Judgments Across All Included Studies.

Domain	Low (%)	Some Concerns (%)	High (%)
Specimen Preparation	100	0	0
Randomization	40	60	0
Blinding	0	100	0
Outcome Measurement	100	0	0
Statistical Analysis	100	0	0
Overall Risk	40	60	0

## Data Availability

The raw data supporting the conclusions of this article will be made available by the authors, without undue reservation.

## Supplementary Materials

The following supporting information can be downloaded at: https://www.mdpi.com/article/10.3390/dj14080513/s1, File S1: PRISMA 2020 checklist.
